# Providers' Knowledge, Attitude and Dispensing Practices of E-Pills in Government Dispensaries of South District in Delhi, India

**DOI:** 10.4103/0970-0218.62553

**Published:** 2010-01

**Authors:** Vertika Kishore, Man M Misro, Deoki Nandan

**Affiliations:** Department of Reproductive Biomedicine, National Institute of Health and Family Welfare, Baba Gangnath Marg, Munirka, New Delhi - 110 067, India

**Keywords:** Emergency pills, health care provider, knowledge, attitude, contraception, dispensing practice

## Abstract

**Background:**

South Delhi is one of the well developed districts in the capital with best public health care facilities. Knowledge, attitude and dispensing practices of emergency contraceptive pills (E-pills) were assessed among health care providers of government dispensaries in South Delhi.

**Study Design:**

A descriptive epidemiological study.

**Materials and Methods:**

Both medical and paramedical (n = 428) providers in 63 government health care facilities were interviewed between August to December 2007 using a semi-structured interview schedule.

**Results:**

Among the different categories of the providers, medical officers were observed to be most knowledgeable about E-pills and the pharmacists were the least. The correct prescribed dose of E-pill was known only to 32% of the providers while 49% knew about its right time of intake. Misconceptions and apprehensions for promoting its use were very much prevalent even among medical officers as majority felt that open access to E-pills would increase promiscuity. The dispensing practice of providers was found positively (*P* < 0.05) correlated with their knowledge. Training resulted a significant (*P* < 0.05) improvement in knowledge, attitude and dispensing practice of the providers. Knowledge and training combined together contributed 35% to the dispensing practice (R^2^ = 0.35).

**Conclusion:**

Besides knowledge, behavior change communication strategies should form a part of the training curricula of health care providers that would help to improve the dispensing practice of E-pills.

## Introduction

The unwanted pregnancy combined with induced unsafe abortions demonstrates the unmet need for emergency contraception. Emergency contraception (EC) refers to contraceptive methods that may be used in the first few days after unprotected sexual intercourse, to prevent unwanted pregnancy. India has the highest number of unsafe abortions in the world. According to government estimates, 8.9% of maternal deaths are caused by unsafe abortions. With a couple protection rate of 56%, the number of unwanted pregnancies is considered reasonably high.(1) In addition, there are accidental pregnancies arising out of condom breakage, miscalculations of safe period, inappropriate coitus interruptus, misplaced intrauterine device and sexual abuse etc.

The socioeconomic burden of unintended pregnancies is significant as most of these end up with unsafe abortions, which would have been otherwise prevented by intervention with ECs. EC is an essential reproductive health option but despite its proven efficacy, it remains vastly underutilized even in a developed country like United States.(2) Patient barriers to use of EC are most notably the lack of awareness among beneficiaries.(3) However, studies which examined knowledge, attitudes and practice patterns of providers like the specialists in Obstetrics and Gynecology and Pediatrics revealed favorable attitudes towards EC, but low rates of provision, knowledge limitations and multiple barriers including fears about patient safety and liability.(4–6) Discrepancies between providers' perceived and actual knowledge have also been reported.(7)

In the Indian context, two recent studies, one from North and another from the East, stated that awareness about EC among paramedicals is abysmally low and practically non-existent. Precise knowledge among doctors including both gynecologists and general practitioners is also inadequate.(8,9) Similar findings were also reported among health workers in Manisa, Turkey, another developing country like India.(10) However, the focus of attention in all these above studies was emergency contraception, which included all the different recognized methods but not the dedicated regimen (levonorgestrel). Government of India approved the dedicated regimen of EC (E-pill) in 2001 and later introduced the same in the National Family Welfare Program in 2003. It was declared an over the counter (OTC) product in September 2005. It is now about five years that the E-pills are being supplied freely to government dispensaries along with other contraceptives, however, information about its dispensing practice among providers in the same dispensaries have never been examined before.

The present study was, therefore, initiated in south district of Delhi to determine the providers' knowledge, attitude and the dispensing practices of E-pills across various government dispensaries.

## Materials and Methods

Out of the nine districts in Delhi, South district was selected since it has the best infrastructure and health care facilities. It has various types of dispensaries under different administrative bodies like Delhi Administration (DA), Municipal Corporation of Delhi (MCD), Central Government Health Scheme (CGHS), Employee State Insurance Corporation (ESIC), India Population Project (IPP-8) etc. In the present study, all dispensaries providing the maternal health and family welfare services were included. Categories of service providers included in the present study were Medical Officer (MO-I/C) or General Duty Medical Officer (GDMO), Staff Nurse (SN), Lady Health Visitor (LHV), Auxiliary Nurse Midwife (ANM) and Pharmacist (PHRM). Efforts were made to include all the providers working in the selected dispensaries. A total of 428 health care providers were covered under the present study.

Primary data was collected from the health care providers during August to December 2007 using a semi-structured interview schedule. The schedule included questions on constituents, dosage, timing and method of intake, side effects, and contraindications of E-pill. Attitude was measured using five-point Likert-type scale.(11) Information on the advance provision of E-pills and imparting counseling at the time of dispension was also collected.

Data were analyzed using SPSS (SPSS-15 version). Statistically significant differences between groups were determined by one way Analysis of Variance (ANOVA). Duncan's means test and f-value was calculated with respect to different variables. Multivariate analysis, appropriate frequency and percentages were calculated wherever necessary. *P* < 0.05 was considered statistically significant.

## Results

South Delhi had 63 health care facilities; out of which 18 belonged to DA, 19 to MCD, 6 to IPP-85, to ESI and 15 to CGHS. MCD had overall administrative, financial and executive control of IPP-8 health care facilities. Among the providers included in the study, 125 were medical officers, 72 Lady Health Visitor/ Staff Nurse, 164 Auxiliary Nurse Midwife (ANMs) and 67 were Pharmacists. A majority (72%) of MOs was females in the age group of 45-55 years (48%). The mean duration of service of MOs was 16 years while two-third of them had in-service experience of more than 15 years. Among 303 paramedical staff (Pharmacists/LHVs/SN/ANMs), 82.5% were females. Male paramedical staff comprised only of pharmacists. Female paramedical staff comprised of 14 pharmacists and the rest (226) consisted of LHV, SN and ANMs. The mean age of paramedical staff was 38 years with majority (43.2%) in the age group of 30-40 years. The mean duration of service for the paramedical providers was 13 years.

The indication for E-pill dispension, most commonly known to providers, was after condom breakage (65.8%) followed by its use in rape victims (49.8%). The less known indications as mentioned were: After expulsion of IUD (16.4%) or delay in receiving monthly dose of contraceptive injection (11.7%). Medical officers demonstrated a better knowledge about the proper indications of use of E-pills. Pharmacists were knowledgeable only in certain aspects like condom breakage (11.9%), miscalculation of fertile period (7.5%), and rape (11.9%). However, they were unaware of the other indications [Table 1].

**Table 1:** Knowledge of health care providers regarding indications of use of E-pills

Provider indications	MO		LHV/SN		ANM		PHARM		Total	
					
	N	%	N	%	N	%	N	%	N	%
Condom breakage	92	73.6	55	76.4	125	76.2	8	11.9	280	65.8
Miscalculation of safe period	57	45.6	17	23.6	29	17.7	5	7.5	108	25.2
Expulsion of IUD	67	53.6	2	2.8	1	0.6	0	0	70	16.4
Failed coitus interruptus	73	58.4	32	44.4	40	24.4	0	0	145	33.9
Failure of OC intake for >3 days	45	36.0	15	20.8	43	26.2	0	0	103	24.1
Late for contraceptive injection	47	37.6	1	1.4	2	1.2	0	0	50	11.7
Rape victims	75	60.0	34	47.2	96	58.5	8	11.9	213	49.8

MO - Medical officer; LHV - Lady health visitor; SN - Staff nurse; PHARM - Pharmacist; N - Number; IUD - Intrauterine device; OC - Oral contraceptive

About half (50.4%) of MOs, a few of LHVs (5.6%) and ANMs (6.1%) knew the correct constituents (levonorgestrel) of the E-pill. But, surprisingly, none of the pharmacists were aware of the composition. The information about the right dose (2 pills) of levonorgestrel (E-pill) was known to 55.2% of MOs, 27.8% of LHV/SNs, 28% of ANMs and only to 7.5% of pharmacists [Table 2]. The correct method of intake of E-pills (2 pills, to be taken at 12 hour interval) was known to 54.4% MOs, 31.9% LHVs/SN, 40.9% ANMs, and 7.5% pharmacists. The time duration within which the pills should be taken (72 hours of unprotected sexual intercourse) was known to 53.6% MOs, 56.9% LHVs/SN, and 57.3% ANMs and to 11.9% pharmacists respectively [Table 2].

**Table 2:** Knowledge among health care providers regarding prescription of E-pills

Provider items	MO		LHV/SN		ANM		PHARM		Total	
					
	N	%	N	%	N	%	N	%	N	%
Constituents of E-pill	63	50.4	4	5.6	10	6.1	0	0	77	18
Prescribed dose	69	55.2	20	27.8	46	28.0	5	7.5	140	32.7
Method of intake	68	54.4	23	31.9	67	40.9	5	7.5	163	38.1
Time duration by which it can be prescribed	67	53.6	41	56.9	94	57.3	8	11.9	210	49.1

MO - Medical officer; LHV - Lady health visitor; SN - Staff nurse; PHARM - Pharmacist; N - Number

**Figure 1:**
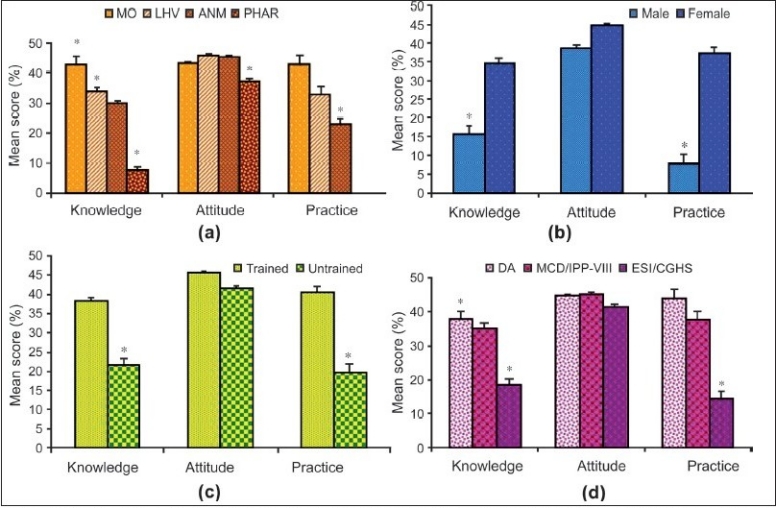
Variations in levels of knowledge, attitude and dispensing practice of E-pills among (a) health care providers (b) Male versus female (c) The impact of training in contraception (d) Working in various dispensaries in south district of Delhi. MO - Medical officer, LHV - lady health visitor, ANM - Auxiliary nurse midwife, PHAR - Pharmacist., DA - Delhi administration, MCD - Municipal corporation of Delhi, IPP - India population project, ESI - Employee's state insurance, CGHS - Central government health service; *P < 0.05

Adverse effects of E-pills were known only to 46.3% of the providers. Almost equal percentages of MOs (61.6%) and LHV/SNs (62.5%) were aware about the same. When further probed, regarding the measures to be taken if a client vomits within two hours of intake of E-pill, 41.4% of the providers responded that they would hardly do anything, 20.3% suggested antiemetics, while 38.3% answered correctly to repeat the dose. Very few providers (7.7%) knew that E-pills are not given to those with confirmed pregnancy. In contrast, 42.8% of the providers were aware that it does not cause any undesirable effects to an ongoing pregnancy. Most of them wrongly considered contraindications such as hypertensive disorders, diabetes, thyroid disorders, heart diseases associated with oral contraceptives as also applicable for E-pills. Most (90%) of the providers had the correct knowledge that E-pills should not be used frequently as this is a back up but not a regular method and need to be used only in emergency. Among the providers, MOs were observed to be most knowledgeable and the pharmacists the least [Figure 1a].

Information gathered from television advertisements, of commercial preparations of EC pills, (48.6% and 38.4%) and that provided by MOs (27.8% and 25.6 %) were the major source of knowledge for the LHV/SNs and ANMs respectively. MOs gathered the knowledge through self-reading either from literature (28.8%), materials received through government channels (22.4%) or trainings (23.2%). Pharmacists gathered the information on E-pills either through self-reading or from TV.

Majority (n=238, 55.6%) of the health care providers, 41.6% of MOs and a higher percentage of paramedics (88.14% LHVs, 84.61% ANMs, and 75% SNs) received training in contraception. The training was imparted under the Reproductive and Child Health (RCH) program of Government of India and was organized by the health agency employing them. Trained providers were found to possess significantly (*P* < 0.05) higher knowledge and attitude that culminated into a better dispensing practice of E-pills than those untrained [Figure 1c]. Pharmacists received no formal training in contraception and were taught about various contraceptive methods only during their teaching curricula, but not updated on this new method of emergency contraception.

Most providers opined that E-pills are easy to use, safe and can prevent pregnancy. However, only 9% felt that its usage would not lead to an increase in promiscuity. Majority had apprehensions that it would be misused once available as an OTC product and therefore had reservations (14%) on advance provisioning of E-pills to clients. Though 85% of the providers [Table 3] advocated counseling for regular family planning methods at the time of dispensing the E-pills, only 30% were found practicing the same [Table 4]. Female providers demonstrated better knowledge, attitude and practice, as they were mostly involved in family planning services [Figure 1b].

**Table 3:** Attitude of the health care providers about E-pills

Item	Strongly agree (%)	Agree (%)	Not sure (%)	Disagree (%)	Strongly disagree (%)
Can prevent unwanted pregnancy	65.9	15.2	19	0	0
Safe and easy administration	59.8	15.7	24.1	0.5	0
Increase promiscuity and misuse	23.6	24.1	43.2	8.2	1
Awareness decreases unsafe abortion	50.5	16.8	30.8	2	0
Advance provision advisable	17.1	12.4	41.4	25.7	3.5
Encourage counseling	68.7	15.9	14	1.5	0
Advocate as OTC product	31.1	14.5	37	15.4	2
Should be followed by regular contraception	66.4	11.7	20	2	0

**Table 4:** Dispensing practice of E-pills among various health care providers*

Provider dispensing practice	MO%	LHV/SN%	ANM%	Total%
Ever dispensed E-pills	59.2	80.6	68.3	57.2
Clients visiting for FW services are made aware about E-pills	45.6	37.5	29.9	31.1
Counseling				
Counsel clients	48.6	31.9	35.4	30.6
Correct counseling	48.0	16.7	28.0	30.1
Advance provision	11.2	13.9	21.3	13.8
Maintaining register	40.8	61.6	57.3	44.2

* Pharmacists are not involved in the dispensing of E-pills; MO - Medical officer; LHV - Lady health visitor; SN - Staff nurse

A similar trend was observed with trained providers who contributed significantly more to the dispensing practice as result of their higher knowledge and favorable attitude than the untrained ones. Among the providers, MOs attitude and dispensing practice completely matched with their knowledge followed by LHV, ANMs. Pharmacists demonstrated the least, as they were not at all involved in the family planning services [Figure 1a]. Providers in the dispensaries where E-pills were available for longer durations had significantly better dispensing practices. Compared to other government dispensaries, providers in the DA dispensaries demonstrated a significantly (*P* < 0.05) better knowledge of E-pills leading to a higher dispensing practice [Figure 1d].

All the three factors, knowledge, attitude and training in contraception showed a positive correlation with the dispensing practice (Significant at *P* value of 0.001) while age and experience a negative correlation [Table 5]. Knowledge about E-pills and training in contraception were seen directly associated (R^2^ = 0.35) with and contributing to 35 % of the dispensing practice of the providers.

**Table 5:** Correlation of dispensing practice with other variables (N = 428)

Indicator	Correlation of coefficient (r)	t-value	β-value
Knowledge	0.5817**	11.1**	0.51
Attitude	0.3053**	0.94	0.04
Age	−0.0006	0.66	0.04
Experience	−0.1002*	−1.34	−0.072
Training in contraception (1 = yes, 0 = no)	0.3282**	2.73**	0.12

N – Number;

** *P* < 0.001;

* *P* < 0.05

## Discussion

Knowledge combined with positive attitude is very much essential to improve the dispensing practice of E-pills. Training emerged as a key component, not only updating the technical knowledge but also gainfully changing the mindset of providers for better dispension of E-pills. E-pills are available free of cost in government dispensaries across the country. However, inadequate knowledge of providers was found directly limiting the dispensing practice of these E-pills in dispensaries of South Delhi. Dispensaries belonging to DA and MCD only were observed to have a better dispensing practice than others [Figure 1d].

MOs were knowledgeable on most aspects of the E-pill, followed by LHVs/ANMs while pharmacists were the least among all the four categories [Tables 1 and 2]. Again, with the exception of pharmacists, knowledge of all categories of providers matched perfectly well with their dispensing practices [Figure 1a]. Knowledge among providers about side effects and failure of E-pills was 46.3% and 42.8% [Data not shown], which were comparatively higher for practice of EC among doctors, which were 15.3% and 35.9% respectively as reported by Mondal *et al*.(9) However, the latter study found the paramedical staff completely unaware of the side effects and the failure of EC. The improvement in knowledge among paramedical providers in the present study might be due to the media campaign (TV and Radio) by commercial firms marketing similar products [Data not shown].

Inadequacies of knowledge about EC among health providers were not specific to India alone. A Nigerian study reported that only 50% of the providers responded correctly to the query on time of intake after unprotected intercourse. However, identical to our findings, the study revealed physicians having a better knowledge and more frequent provision of EC than nurses, pharmacists and community health workers.(12) The health care workers in Turkey showed similar gaps in knowledge about EC and hesitant attitudes while practicing the same.(10)

Even in University of Michigan, USA, one-third of all the respondents consisting of faculty, residents and nurses in the department of family medicine were unaware of the correct time interval of initiating EC.(7) Thus, barriers to EC use was not only limited to social, religious or ethical beliefs but also to a greater extent to suboptimal knowledge level of those responsible for regular and proper dispensing of the same.

In the present study, pharmacists were found not involved in any kind of family welfare activities. This may be due to their ever-increasing workload of dispensing all kinds of drugs but not contraceptives for patients in general out patient departments (OPDs). Pharmacists constitute a major category of health care providers in any health setup and their selective engagement and work assignment had poorly reflected upon their knowledge on E-pills. In our study, 12% of pharmacists were aware about different aspects of E-pills compared to 25% having some idea about ECs.(13) Gaps in the knowledge among pharmacists on emergency contraception were also reported from South Dakota, USA; it was suggested that pharmacists should be provided with opportunities for practice counseling on ECs in the form of both formal and continuing education training.(14)

Providers with training in contraception were found to possess better knowledge and attitude than those not trained [Figure 1c]. Again, females fared better than males in this regard [Figure 1b]. This is more to do with Indian social system and the prevalent inhibition leading to preference among female patients consulting female health providers. But the training had a better impact on the dispensing practice irrespective of sex. The pharmacists were never sent for any training in contraception as their present duty profile did not necessitate such a demand. Training from MOs and through self reading were the important source of knowledge for more than 50% of the existing paramedical catering to family welfare services [Data not shown].

Misconceptions and apprehensions reflect the negative attitude of providers.(15) In the present study, the attitudes of various categories of providers towards E-pills were positive and, most of the time, either matching with their level of knowledge or even sometimes more than the perceived knowledge. But, in a real situation, it is the knowledge that brings a pivotal role in decision making as positive attitude with inadequate knowledge does not commensurate at all with the level of dispensing practice [Figure 1].

About 55% of the medical officers in the present study stated that E-pill usage would increase promiscuity and misuse among the users, especially the younger generation and thus disagreed for its advance provisioning and availability as an OTC product. Only about 10% demonstrated a positive attitude that if used correctly, it would not increase the misuse [Table 3]. The present findings are in conformity with many such similar studies, which revealed the general feeling from many health professionals that easy access to EC would encourage unsafe sexual relations and discourage the use of more reliable contraception.(16,17)

## Conclusions

It is evident that for efficient dispensing practice of E-pills, the correct knowledge along with the positive attitude is very much essential. This could be achieved through training strategies aimed to improve not only the technical knowledge but also the behavior change communication, which is essential to positively modulate the mindset of the providers in a scientific way. In addition, the role of pharmacists as health care provider vis-à-vis family welfare services needs to be reassessed again by policy makers and program managers in order to fully make use of their skill and potential.
